# Potentiation of Glucose-stimulated Insulin Secretion by the GPR40–PLC–TRPC Pathway in Pancreatic β-Cells

**DOI:** 10.1038/srep25912

**Published:** 2016-05-16

**Authors:** Hodaka Yamada, Masashi Yoshida, Kiyonori Ito, Katsuya Dezaki, Toshihiko Yada, San-e Ishikawa, Masafumi Kakei

**Affiliations:** 1 First Department of Comprehensive Medicine, Jichi Medical University Saitama Medical Center, Amanuma, Omiya 1-847, Saitama 330-8503, Japan; 2 Division of Integrative Physiology, Department of physiology, Jichi Medical University School of Medicine, Yakushiji 3311-1, Shimotsuke, Tochigi 329-0498, Japan

## Abstract

G protein-coupled receptors (GPCRs) are expressed in pancreatic beta-cells. G protein-coupled receptor 40 (GPR40) contributes to medium- or long-chain fatty acid-induced amplification of glucose-stimulated insulin secretion (GSIS), and GPR40 agonists are promising therapeutic targets in type 2 diabetes. Recently, we demonstrated that glucagon-like peptide 1, a ligand of pancreatic GPCR, activates a class of nonselective cation channels (NSCCs) and enhances GSIS. The aim of the current study was to determine whether the GPR40 signal interacts with NSCCs. A GPR40 agonist (fasiglifam) potentiated GSIS at 8.3 and 16.7 mM glucose but not 2.8 mM glucose. The NSCC current was activated by fasiglifam at 5.6 mM glucose with 100 μM tolbutamide (−70 mV), and this activation was prevented by the presence of pyrazole-3 (transient receptor potential canonical; a TRPC3 channel blocker). Inhibitors of phospholipase C or protein kinase C (PKC) inhibited the increases in GSIS and the NSCC current induced by GPR40 stimulation. The present study demonstrates a novel mechanism for the regulation of insulin secretion by GPR40 agonist in pancreatic beta-cells. The stimulation of the GPR40–PLC/PKC–TRPC3 channel pathway potentiates GSIS by the depolarization of the plasma membrane in pancreatic beta-cell.

G protein-coupled receptors (GPCRs) are an important target of innovative drug development for type 2 diabetes^1^. G protein-coupled receptor 40 (GPR40) is highly expressed in pancreatic beta-cell^2^,^3^, and its agonistic stimulation enhances glucose-stimulated insulin secretion (GSIS), thereby being a promising therapeutic target in type 2 diabetes^4^,^5^,^6^. GPR40 is a Gq-coupled protein receptor, and its ligands are unsaturated medium- or long-chain free fatty acids. Stimulation of GPR40 signal activates phospholipase C (PLC). PLC hydrolyzes phosphatidylinositol 4,5-bisphosphate (PIP_2_), resulting in production of inositol 1,4,5-trisphosphate (IP_3_) and diacylglycerol (DAG). Increased IP_3_ binds to the IP_3_ receptor of the endoplasmic reticulum (ER) and mobilizes Ca^2+^ to increase intracellular Ca^2+^ concentration ([Ca^2+^]_i_) from the ER^1^,^7^,^8^,^9^,^10^,^11^. DAG promotes F-actin remodeling and potentiates GSIS via protein kinase D1^12^. Incretin hormone glucagon-like peptide 1 (GLP-1) or glucose-dependent insulinotropic polypeptide (GIP), a ligand of the Gs-coupled protein receptor of pancreatic beta-cell, stimulates adenylate cyclase and increases cytosolic cyclic adenosine 3′,5′-monophosphate (cAMP). Produced cAMP enhances the activity of protein kinase A (PKA) and exchange protein directly activated by cAMP 2 (EPAC2)^13^. Recently, we demonstrated that the cAMP–EPAC2 pathway increases [Ca^^2^+^]_i_ via membrane depolarization as a consequence of openings of the nonselective cation channel (NSCC) transient receptor potential melastatin 2 (TRPM2)^14^. TRPM2 is expressed in rats and mice pancreatic beta-cell^15^, and TRPM2-knockout mice showed the impairment of glucose- and GLP-1-mediated insulin secretion^16^. We have reported that glucose and GLP-1 increase NSCC currents and cooperatively facilitate the depolarization of beta-cell membranes with the glucose-induced closure of ATP-sensitive potassium (KATP) channels^14^. However, whether the GPR40 agonist regulates NSCC activity remains unclear. The aims of the current study are to determine the following: 1) whether the GPR40 signal interacts with the NSCC current, 2) the mechanistic pathway downstream of GPR40 stimulation, and 3) what type of NSCC is identified if NSCCs are involved in the pathway. It is known that fasiglifam is a selective and highly bioavailable GPR40 agonist^17^. In this study, we used fasglifam.

## Results

### Fasiglifam, a GPR40 agonist, depolarizes the plasma membrane and increases background current in pancreatic beta-cell

**Figure 1:**
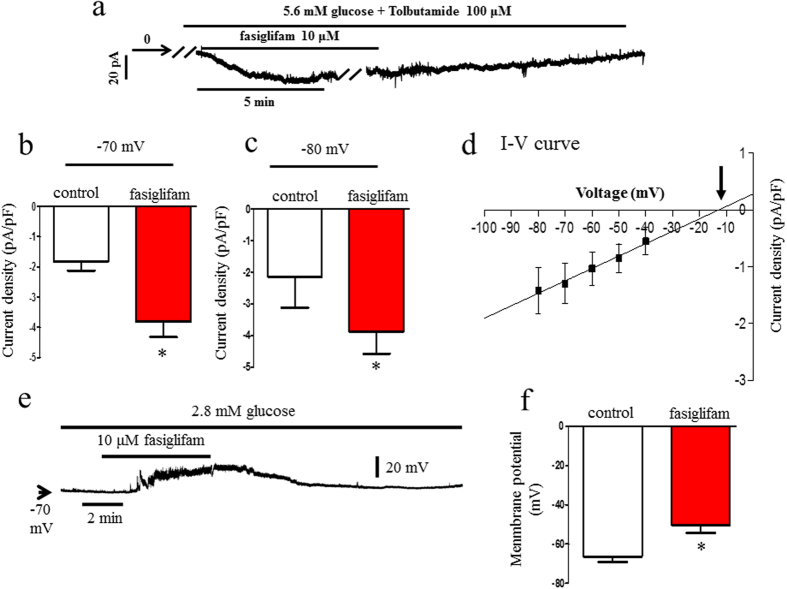
Fasiglifam increased nonselective cation channel (NSCC) current and depolarized the resting membrane potential in pancreatic rat single beta-cell. (**a**) Effect of fasiglifam on the holding current recorded from a beta-cell. The cell was superfused with 5.6 mM glucose and 100 μM tolbutamide at the holding potential of −70 mV. The holding current was increased for the inward direction in the presence of 10 μM fasiglifam. (**b**) The current density that was expressed as pA/pF (amplitude of the holding current was divided by membrane capacitance that was measured on the establishment of whole-cell clamp) was shown, and fasiglifam significantly increased the current density. (**c**) Fasiglifam-induced increase in NSCC current was observed in the absence of tolbutamide at a holding potential of −80 mV. (**d**) The current–voltage relationship showed a reversal potential of −12 mV (arrow). The line was drawn using linear regression fit to the data points. (**e**) The membrane potential was recorded at 2.8 mM glucose. The membrane was depolarized upon superfusion with 10 μM fasiglifam in a reversible manner. (**f** ) Comparison of resting membrane potentials in the absence and presence of 10 μM fasiglifam. The glucose concentration was 2.8 mM. Membrane potentials in the absence and presence of 10 μM fasiglifam were −66.5 ± 2.9 and −50.5 ± 3.7 mV, respectively. Data were expressed as mean ± standard error of the mean. The number of data points was five. *P < 0.05 vs. control by paired t-test.

First, to test the effects of fasiglifam on the NSCC current without the influence of changes in activity of the KATP channel by fasiglifam, we voltage-clamped the cells at −70 mV, which is close to the potassium equilibrium potential, and used tolbutamide to inhibit the KATP channel at 5.6 mM glucose (Fig. 1a,b). In these situations, the influence of the KATP channel on the NSCC current is negligible. Fasiglifam significantly increased the inward current in a reversible manner. To eliminate the influence of tolbutamide, we examined the current recorded at the holding potential of −80 mV in the absence of tolbutamide, which is closer to the potassium equilibrium potential of −82 mV when we assumed an intracellular potassium concentration of 140 mM with a 5 mM K^+^ extracellular solution. At the holding potential, the NSCC current was increased (Fig. 1c), suggesting that tolbutamide was not related to the NSCC-current increase by fasiglifam. Figure 1d shows the current–voltage relationship that was constructed by subtracting a series of currents induced by voltage steps in the absence of fasiglifam from those recorded during the exposure of the cell to fasiglifam, as shown in Supplementary Fig. 1. The current–voltage relationship of the subtracted currents reversed at −12 mV. These results indicate that the fasiglifam-induced current was due to the activation of NSCC. The reversal potentials were consistent with NSCC reversal potentials in previous reports (reversal potentials ranging from −20 to 0 mV; Fig. 1d)^18^. The NSCC current is voltage-independent (Supplementary Fig. 1).

We next examined whether the fasiglifam-induced inward current affects the membrane potential. The plasma membrane was reversibly depolarized during the exposure of the beta-cells to 10 μM fasiglifam at 2.8 mM glucose. However, fasiglifam-induced depolarization did not reach a threshold potential sufficient to evoke the action potential (Fig. 1e). Membrane potentials in the absence and presence of 10 μM fasiglifam were −66.5 ± 2.9 mV and −50.5 ± 3.7 mV (P = 0.0005), respectively (Fig. 1f). Because the membrane potential is regulated by a balance between KATP-channel current and NSCC current, we further performed the experiments under the condition of sub-threshold concentration to trigger action potential firing (Supplementary Fig. 2). The plasma membrane was mildly depolarized at 5.6 mM glucose and after the exposure of the beta-cell to 10 μM fasiglifam the action potentials were initiated. To distinguish whether the fasiglifam-induced depolarization is due to KATP-channel inhibition, we further performed the experiments under the condition of current injection to hyperpolarize the membrane and inhibit action potentials elicited by 100 μM tolbutamide. In this situation, the KATP-channel activity was inhibited (Supplementary Fig. 3). The firing of action potentials was evoked again during 10 μM fasiglifam exposure. Thus, fasiglifam-induced depolarization was independent of KATP channel activity.

**Figure 2:**
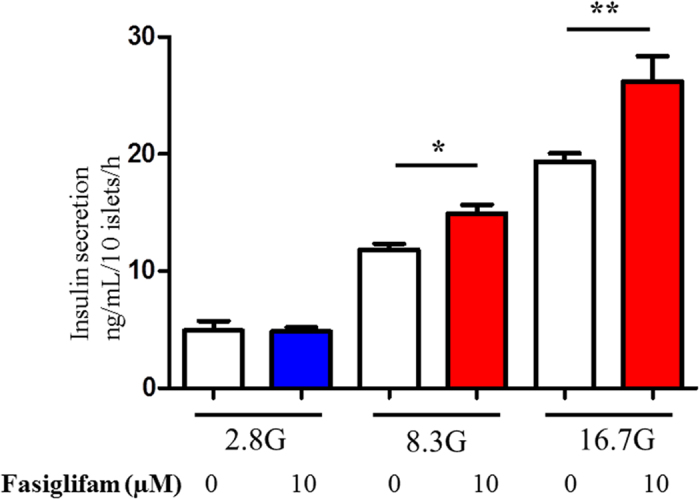
Fasiglifam potentiated insulin secretion at supra-threshold concentration of glucose. Each batch containing 10 size-matched rat islets was incubated for 1 h to evoke insulin secretion. Fasiglifam potentiated insulin secretion at 8.3 and 16.7 mM glucose. However, fasiglifam did not potentiate insulin secretion at 2.8 mM glucose. The number of data points was five to seven. *P < 0.05 vs. 8.3 mM glucose; **P < 0.01 vs. 16.7 mM glucose by unpaired t-test.

Insulin secretion stimulated by 8.3 or 16.7 mM glucose was potentiated by the addition of 10 μM fasiglifam but not by 2.8 mM glucose (Fig. 2). Although we have measured GSIS from 10 size-matched islets that were collected with picking by hand, consistent results were confirmed by normalizing insulin secretion with protein content as revealed in Supplementary Fig. 4. Fasiglifam over 0.1 μM dose-dependently increased insulin secretion in rat islets compared with that in the presence of 16.7 mM glucose (Supplementary Fig. 5). Potentiation of GSIS by fasiglifam was also observed in mouse islets (Supplementary Fig. 6). Stimulatory effects of fasiglifam on insulin secretion are similarly observed in both mouse and rat islets.

### Fasiglifam increased the NSCC current via the PLC/PKC pathway

**Figure 3:**
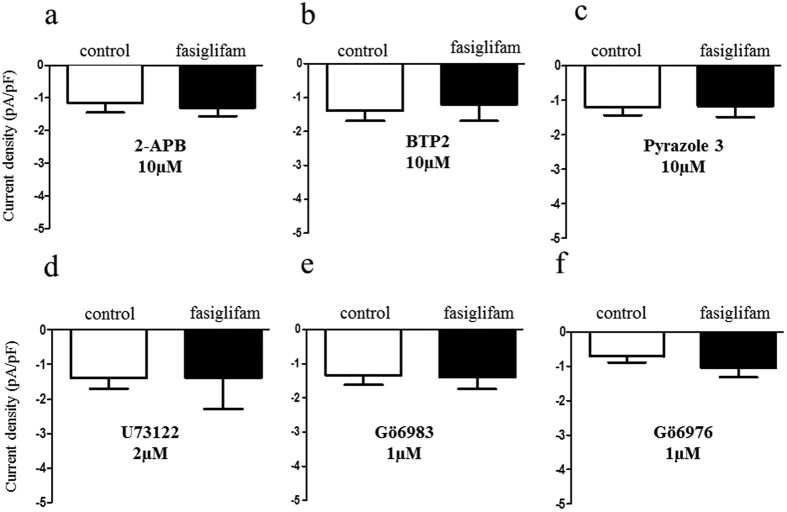
Abolition of fasiglifam-induced increase in nonselective cation channel (NSCC) current by inhibitors of the phospholipase (PLC)/protein kinase C (PKC) and TRP channels. (**a–c**) Effects of the nonselective transient receptor potential (TRP) channel blocker 2-aminoethyl diphenylborinate (2-APB; 10 μM) (**a**), TRP canonical (TRPC) channel blocker 3,5-bis(trifluoromethyl)pyrazole derivative 2 (BTP2; 10 μM) (**b**) and selective TRPC3 channel blocker pyrazole-3 (Pyr3; 10 μM) (**c**) on the NSCC current. (**d–f**) Effects of the PLC inhibitor U73122 (2 μM) (**d**) and PKC inhibitors Gö6983 (1 μM) (**e**) and Gö6976 (1 μM) (**f**) on the NSCC current. Fasiglifam-induced current increases were inhibited by these inhibitors. Rat single beta cells were voltage-clamped at −70 mV in the presence or absence of 10 μM fasiglifam, under the condition of 5.6 mM glucose and 100 μM tolbutamide throughout the experiments. The number of data points was five.

**Figure 4:**
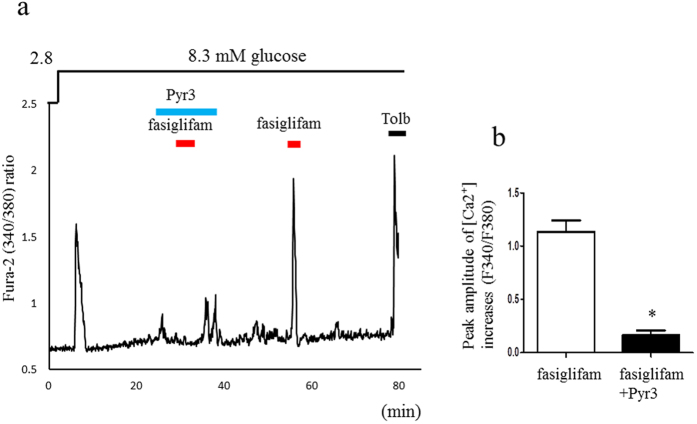
Transient receptor potential canonical 3 (TRPC3) channel blocker inhibited fasiglifam-induced [Ca^2+^]_i_ increase. (**a**) Fasiglifam (10 μM) increased intracellular Ca^2+^ concentration ([Ca^2+^]_i_) and pyrazole-3 (Pyr3, 10 μM) inhibited fasiglifam-induced [Ca^2+^]_i_ increase at 8.3 mM glucose. At the end of the experiment, 100 μM tolbutamide (Tolb) was added to confirm that the responsive cells were beta-cell. (**b**) Pyr3 at 10 μM attenuated fasiglifam-induced [Ca^2+^]_i_ increases in single mouse beta-cell. (*n* = 16, number of single mice beta-cells examined in each group); ^∗^P < 0.01 vs. fasiglifam.

**Figure 5:**
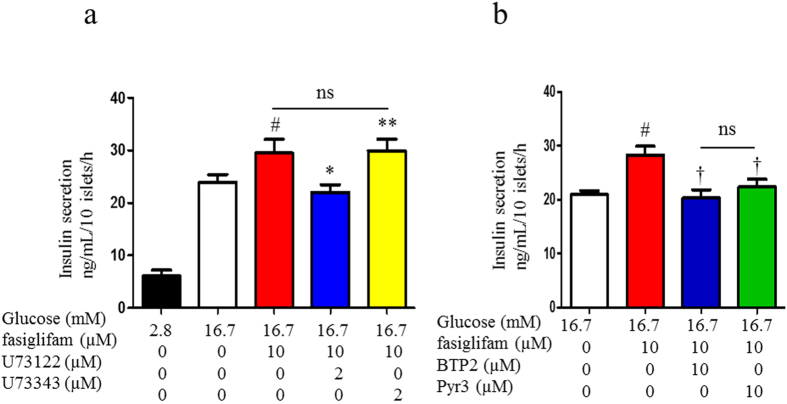
Phospholipase C (PLC) inhibitor and transient receptor potential canonical (TRPC) channel blocker inhibited fasiglifam-induced insulin secretion. (**a**) Batches containing 10 rat islets in each were incubated for 1 hour to evoke insulin secretion at 2.8 or 16.7 mM glucose. Addition of fasiglifam (10 μM) potentiated insulin secretion at 16.7 mM glucose. The PLC inhibitor U73122 inhibited fasiglifam-induced insulin secretion at 16.7 mM glucose. U73443, a negative control for PLC inhibition, did not affect fasiglifam-mediated insulin secretion. (**b**) TRPC channel blocker 3,5-bis(trifluoromethyl)pyrazole derivative 2 (BTP2) and TRPC3 channel selective blocker pyrazole-3 (Pyr3) inhibited fasiglifam-induced insulin secretion at 16.7 mM glucose. The number of data points was four to 14. *P < 0.05 vs. 10 μM fasiglifam at 16.7 mM glucose; ^†^P < 0.01 vs. 10 μM fasiglifam at 16.7 mM glucose ; ^#^P < 0.05 vs. 16.7 mM glucose; **P < 0.05 vs. 10 μM fasiglifam at 16.7 mM glucose with 2 μM U73122 by unpaired t-test. ns; not significant between two groups.

We reported that the NSCC current was increased in Ca^2+^ influx via a class of TRP channel (TRPM2) by the incretin hormones GLP-1 and GIP^14^. We examined whether the TRP channel blockers 2-aminoethyl diphenylborinate (2-APB), a nonselective TRP channel blocker and 3,5-bis(trifluoromethyl)pyrazole derivative 2 (BTP2), a selective TRP canonical (TRPC) channel blocker, inhibit the NSCC-current increase induced by fasiglifam (Fig. 3a,b). Both these blockers attenuated the NSCC current induced by fasiglifam. Pyrazole-3 (Pyr3), a selective blocker for the TRPC3 channel, also inhibited the NSCC current increase induced by fasiglifam (Fig. 3c and Supplementary Fig. 7 for original current trace). These results suggest that fasiglifam activates the TRPC3 channel and thereby increases the NSCC inward current. Previous reports indicate that PLC activity is involved in a pathway downstream of GPR40 stimulation^9^,^19^. We examined whether the PLC inhibitor U73122 affects the NSCC-current increase evoked by fasiglifam. U73122 at 2 μM inhibited the NSCC-current increase by fasiglifam (Fig. 3d). Two different types of blockers for PKC inhibited the fasiglifam-induced NSCC current (Fig. 3e,f). Next, we examined whether fasiglifam-induced increase in [Ca^2+^]_i_ was influenced by Pyr3 treatment by using dual-wavelength fura-2 microfluorometry. In the presence of 8.3 mM glucose, 10 μM fasiglifam increased [Ca^2+^]_i_ in mouse single beta-cells (Fig. 4a). This [Ca^2+^]_i_ increases were similarly observed in rat beta-cell (Supplementary Fig. 8). On the other hand, the peak amplitude of fasiglifam-induced [Ca^2+^]_i_ increases was significantly suppressed by pretreatment with 10 μM Pyr3 (Fig. 4a,b). In an insulin release assay (batch incubation), GSIS with 16.7 mM glucose was significantly enhanced by co-incubation with fasiglifam. Blockade of PLC by U73122 abolished the fasiglifam-potentiated GSIS (Fig. 5a). Blockades of the TRPC channel by BTP2 and the TRPC3 channel by Pyr3 also inhibited fasiglifam-potentiated GSIS (Fig. 5b).

### The cAMP pathway is not involved in the potentiation of insulin secretion by fasiglifam

**Figure 6:**
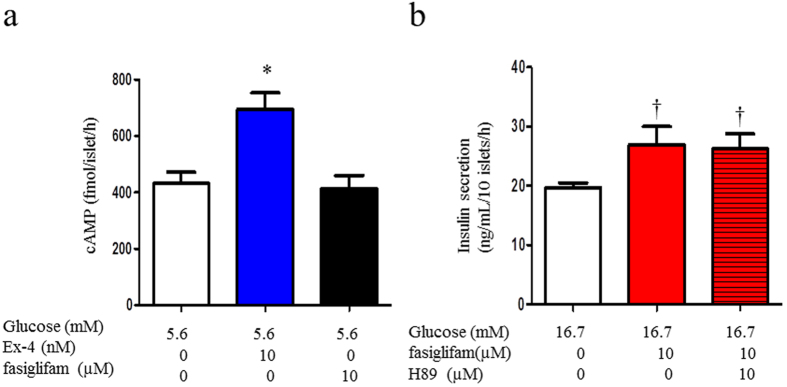
The cAMP–PKA pathway was not involved in fasiglifam-evoked insulin secretion. (**a**) The cAMP production induced by 5.6 mM glucose was potentiated by exendin-4 (10 nM) in isolated islets. Fasiglifam (10 μM) did not significantly affect the cAMP production induced by 5.6 mM glucose. Batches containing 10 rat islets in each were incubated for 1 hour at 5.6 mM glucose. The number of data points was three. (**b**) The protein kinase A (PKA) inhibitor H89 (10 μM) did not affect fasiglifam-induced insulin secretion. Batches containing 10 rat islets in each were incubated for 1 h to evoke insulin secretion at 16.7 mM glucose. The number of data points was five. *P < 0.05 vs. 5.6 mM glucose; ^†^P < 0.01 vs. 16.7 mM glucose by unpaired t-test.

Because the cAMP/PKA and cAMP/EPAC pathways play an important role in the insulin secretion mechanism of beta-cell, we examined whether GPR40 signaling interacts with the cAMP pathway. Exendin-4 increased cytosolic cAMP levels in isolated rat islets at 5.6 mM glucose, whereas fasiglifam did not increase cAMP levels (Fig. 6a). Furthermore, H89, a PKA inhibitor, did not affect fasiglifam-induced GSIS (Fig. 6b). These results suggested that the cAMP/PKA pathway did not interact with the GPR40 signaling pathway. Recently, we reported that incretin and glucose metabolism activates the cAMP/EPAC/TRPM2 pathway and potentiates insulin secretion^14^. The TRPM2 channel, which is activated by GLP-1 at a concentration of ≥100 pM or by glucose >5.6 mM, cooperatively depolarizes the membrane in concert with the closure of KATP channels as a consequence of glucose metabolism. The activation of the NSCC current by glucose or GLP-1 was not observed in TRPM2-deficient mice^14^. We examined whether fasiglifam activates the NSCC current via TRPM2 channels in beta-cells from C57BL/6J mice and TRPM2-knockout mice at 5.6 mM glucose. In wild-type and TRPM2-knockout mice, the NSCC current was similarly increased by fasiglifam (Supplementary Fig. 9a,b).

## Discussion

It is well known that free fatty acid receptor binding to GPR40 stimulates PLC, resulting in the production of DAG and IP_3_ from PIP_2_ hydrolysis. In this study, we found that GPR40 stimulation increases the NSCC current because of openings of TRPC3 channels and depolarizes the membrane followed by action potential firings in cooperation with KATP channel closure over sub-threshold concentrations of glucose. These multiple and simultaneous stimulations of molecules required for initiation of insulin secretion effectively potentiate GSIS compared with glucose-induced KATP channel inhibition pathway alone. These findings demonstrated for the first time that TRPC3 activation is a novel molecular mechanism of GSIS potentiation by the GPR40 agonist.

Various types of TRP channels, TRPA1, TRPC1, TRPC4-C6, TRPV1, TRPV2-V5, and TRPM2-M5, are reportedly expressed in pancreatic beta-cell^20^. We also confirmed the expression of TRPC3 in rat and mouse pancreatic beta-cells (Supplementary Fig. 10). Fasiglifam, an agonist of GPR40, increased the NSCC current via the TRPC channel.The findings of previous reports that PLC inhibition abolished oleic acid-mediated GSIS^9^,^19^ and of our study showing the attenuation of fasiglifam-potentiated GSIS and fasiglifam-induced inward current via PLC inhibition by U73122 are consistent. DAG and IP_3_ produced by PLC stimulation affects receptor-operated TRP channels and store-operated TRP channels^21^. The store-operated mechanism has recently been regarded as the stromal interaction molecule 1 (STIM1)-Orai1 system^22^,^23^. PLC inhibition abrogated the NSCC current mediated by fasiglifam, suggesting that PLC is one of the upstream molecules that transduce some signals to TRPC on the plasma membrane. Further studies are needed to clarify the mechanisms downstream of the GPR40–PLC pathway.

GPR40 signaling did not affect the cAMP-dependent pathway in our results. The cAMP-dependent pathway is an important pathway to promote insulin secretion by incretins GLP-1 and GIP. We recently reported that GLP-1 increased the NSCC current via the TRPM2 channel activation in rat and mouse pancreatic beta-cell^14^. The GLP-1 receptor is a Gs-coupled receptor, and its stimulation induces adenylate cyclase activation and cAMP production, which activates PKA and EPAC^1^. The cAMP–EPAC–TRPM2 pathway signaling opens TRPM2 channels and induces [Ca^2+^]_i_ increase via the membrane depolarization. These lead to further depolarization of the cell membrane in cooperation with the closure of the KATP channel that is mediated by glucose metabolism and ATP/ADP elevation at cytosol. The present study revealed that the PKA inhibitor H89 had no effect on the fasiglifam-induced current increase in NSCC, and the inward current increases were observed in TRPM2-knockout mouse primary beta-cells (Supplementary Fig. 9). Although exendin-4 increased cytosolic cAMP concentration, fasiglifam did not affect the cAMP concentration in rat islets. These results suggested that the GPR40–PLC-mediated mechanism of insulin secretion is a cAMP-independent pathway in pancreatic beta-cell. There are some reports that GPR40 stimulation by its agonist and free fatty acids did not increase cytosolic cAMP in mouse islets and cultured cells^24^,^25^,^26^,^27^. Our study supports these previous reports. However, another study showed that a GPR40-mediated insulinotropic mechanism depends on the opening of L-type Ca^2+^ channels (LTCCs). Fujiwara *et al*. reported that GPR40 stimulation by oleic acid enhanced Ca^2+^ influx from outside of the plasma membrane via LTCCs that opened at glucose concentrations over threshold in rat pancreatic beta-cell^9^. An LTCC blocker inhibited GPR40-mediated insulin secretion in rat islets and INS-1E cells^9^,^19^,^27^. It was then pointed out that KATP channels not only provide an interface of metabolic changes with electrical excitation but also rapidly transmit extracellular signals through GPCRs and the phosphatidylinositol–PLC pathway via PIP_2_ metabolism^28^. It is reported that the pharmacological inhibition of LTCC prevented the Ca^2+^ increase caused by GPR40 stimulation with oleic acid^9^. These findings suggest that the GPR40-induced Ca^2+^ increase is mediated by Ca^2+^ influx through the plasma membrane presumably via LTCC rather than via IP_3_-mediated Ca^2+^ efflux from the ER. Thus, the IP_3_ signal plays a rather minor role in cytosolic Ca^2+^ concentration. Ca^2+^ influx from outside of the plasma membrane is more important^29^. There is a possibility that PIP_2_ depletion or increased DAG induced by GPR40-PLC signaling directly affects TRPC3 activation. An interaction between LTCC and TRPC also remains to be elucidated.

PKC is involved in fatty acid-induced insulin secretion. Some reports suggested that general PKC inhibitors abolished fatty acid-induced insulin secretion^30^. The current study showed the suppression of increases in the inward current by treatment with general PKC inhibitors, suggesting that PKC activated NSCCs and increased [Ca^2+^]_i_. Pancreatic beta-cell have complex mechanisms to increase [Ca^2+^]_i_.

Shigeto *et al*. reported that a mechanism for picomolar levels (1–10 pM) of GLP-1 effect on GSIS that involves the activation of Gq/PLC/PKC pathway but no cAMP and the increase of inward current because of TRPM4/TRPM5 activations, Na^+^-permeable channels in pancreatic beta-cell^31^. TRPM4/M5 channels were opened by the mobilization of intracellular Ca^2+^ from thapsigargine-sensitive Ca^2+^ store. Their findings of GLP-1 < 10 pM work as an activator of TRPM4/5 channels. Thus, TRPM4/5 channels open at basal condition because such a low concentration of GLP-1 is identical to that before meal stimulation. Whether both the low-dose GLP-1-dependent signaling pathway and GPR40-stimulation pathway revealed in the present study use the same Gq/PLC/PKC molecules as a common pathway or these pathways are separately coupled to TRPM4/M5 and TRPC3 are unknown. Further studies are needed to clarify the relationship between the Gq-coupled GPCR and the NSCC current.

In summary, the present study places TRPC in the GPR40 signaling pathway in pancreatic beta-cell and describes the GPR40–PLC/PKC–TRPC3 pathway as a novel cooperator of the KATP channel closure in the depolarization of beta-cell. The current study showed that TRP channel family activation is a promising therapeutic target for diabetes.

## Methods

### Preparation of islets and single pancreatic beta-cell

Male Wistar rats and C57BL/6J mice (CLEA Japan, Inc.) were housed in accordance with our institutional guidelines and the Japanese Physiological Society’s guidelines for animal care in an air-conditioned room with a 12-h light/dark cycle, and food and water were available *ad libitum*. Islets of Langerhans were isolated by collagenase digestion from male Wister rats (aged 8–12 weeks) and C57BL/6J mice using a previously reported method^32^,^33^. Briefly, animals were anesthetized by the intraperitoneal injection of pentobarbital (100 mg/kg), followed by the injection of collagenase (Sigma-Aldrich, Tokyo, Japan), 1.05 mg/mL, dissolved in HEPES-added Krebs-Ringer bicarbonate buffer (HKRB) containing 5 mM CaCl_2_ directly into the common bile duct. Ordinal compositions of HKRB were 129 mM NaCl, 5 mM NaHCO^3^, 4.7 mM KCl, 1.2 mM KH_2_PO_4_, 2 mM CaCl_2_, 1.2 mM MgSO_4_, and 10 mM HEPES, at pH 7.4 with NaOH. The pancreas was dissected out and incubated at 37 °C for 15 min in the HKRB buffer. Size-matched islets were collected and used for insulin release experiments in static incubation. In addition, electrophysiological experiments using single beta-cell dispersed in Ca^2+^-free HKRB were performed. HKRB with 0.01% bovine serum albumin (fatty acid free; purchased from Sigma-Aldrich) was used for insulin secretion measurements. All experimental protocols for animal studies were approved by the institutional committee on animal care in Jichi Medical University.

### Measurements of insulin secretion from rat islets and cAMP

Each batch of size-matched 10 islets was incubated for 1 h at 37 °C in Ca^2+^-free HKRB with 2.8 mM glucose for stabilization, followed by incubation for 1 h in HKRB with 2.8 or 16.7 mM glucose with and without fasiglifam (10 μM; AdooQ BioScience). Other compounds were added to test incubation batches at various concentrations, as described below. BTP2 (10 μM; Cayman Chemical, Ann Arbor, MI, USA) and Pyr3 (10 μM; Sigma-Aldrich, St. Louis, MO, USA) were used as TRP channel blockers. After 60 min incubation in HKRB with 2.8 or 16.7 mM glucose with fasiglifam and BTP2, Pyr3, U73122 (a phospholipase C inhibitor, 2 μM; Sigma-Aldrich), U73343 (a negative control for PLC inhibitor, 2 μM; Sigma-Aldrich), and H89 (a PKA inhibitor, 10 μM; Sigma-Aldrich), secreted insulin concentrations in the supernatants of each test batch were determined using ELISA kits (Morinaga Institute of Biological Science, Yokohama, Japan). For the cAMP assay, each tube containing 10 islets was incubated for 60 min in HKRB with 5.6 mM glucose containing 500 μM 3-isobutyl-1-methylxanthine, an inhibitor of phosphodiesterase, and the cAMP level was determined using an enzyme immunoassay kit (GE Healthcare, Buckinghamshire, UK)^34^.

### Measurement of cytoplasmic Ca^2+^ concentration

Single beta-cells were isolated from male C57BL/6J mice and plated on coverslips. Cytoplasmic Ca^2+^ concentration ([Ca^2+^]_i_) in beta-cells was measured as previously reported^14^,^33^. Briefly, beta cells were superfused with HKRB at 36 °C, and [Ca^2+^]_i_ was measured by dual-wavelength fura-2 microfluorometry with excitation at 340/380 nm and emission at 510 nm, using a cooled charge-coupled device camera. Fluorescence ratio images were produced using an Aquacosmos system (Hamamatsu Photonics, Hamamatsu, Japan). Cells used for single-cell experiments fulfilled the morphological and physiological criteria for insulin-positive beta-cell, including the diameter and responsiveness to glucose and tolbutamide. Effects of fasiglifam on [Ca^2+^]_i_ were investigated exclusively in the cells that responded to glucose with increases in [Ca^2+^]_i_ in a beta-cell-specific manner and to tolbutamide at the end of recording.

### Electrophysiological experiments

Perforated whole-cell currents were recorded using a pipette solution containing amphotericin B (200 μg/mL) dissolved in 0.1% dimethylsulfoxide. Membrane currents, recorded using an amplifier (Axopatch 200B; Axon Instruments, Foster City, CA), were stored online in a computer with pCLAMP10.2 software. The voltage clamp in perforated mode was considered to be adequate when the series resistance was <20 MΩ. Patch pipettes purchased from Narishige (Tokyo, Japan) and their resistances ranged from 3 to 5 MΩ when filled with pipette solution that contained 40 mM K_2_SO_4_, 50 mM KCl, 5 mM MgCl_2_, 0.5 mM EGTA, and 10 mM HEPES at pH 7.2 with KOH. HKRB solution containing 5.6 mM glucose was used as the external solution. For recording of the NSCC current, rat or mouse single beta-cells were voltage clamped at a holding potential of −70 mV in the presence of 100 μM tolbutamide, which is a sufficient concentration to specifically block KATP channels. Thus, the residual current is the NSCC current. Electrophysiological experiments were performed at 27 °C^14^. To avoid the possibility of a tiny influence of fasiglifam or tolbutamide on the KATP channel current that may produce errors against the results regarding the NSCC current evaluations, the perforated whole-cell current was recorded at the holding potential of −80 mV in the absence of tolbutamide because this potential is identical to the potassium equilibrium potential, and the KATP channel current at that potential should be negligibly small. Measurement of membrane potentials was performed by switching from perforated whole-cell voltage-clamp mode to current-clamp mode. Current was injected to hyperpolarize the membrane that was depolarized by tolbutamide to evaluate effects of fasiglifam on the membrane potential regardless of KATP-channel activity. HKRB solution containing 2.8 or 5.6 mM glucose was used for external solution. We demonstrated that the voltage-clamped cell was immunostained with anti-insulin antiserum and shown to express insulin-positive fluorescence^14^. Electrophysiological experiments were performed at 27 °C. 2-APB (10 μM; Wako), BTP2, and Pyr3 were used as TRP channel blockers. Gö6983 (1 μM; Wako) and Gö6976 (1 μM; Merck Millipore) were used for blocking PKC.

### Statistical Analysis

Data are presented as mean ± standard error of the mean and were compared using Student’s t-test performed with GraphPad Prism version 5.0. P values of <0.05 were considered statistically significant.

## Additional Information

**How to cite this article**: Yamada, H. *et al*. Potentiation of Glucose-stimulated Insulin Secretion by the GPR40–PLC–TRPC Pathway in Pancreatic β-Cells. *Sci. Rep.* **6**, 25912; doi: 10.1038/srep25912 (2016).

## Supplementary Material

Supplementary Information
