# Supplementary material for: Potentiation of Glucose-stimulated Insulin Secretion by the GPR40–PLC–TRPC Pathway in Pancreatic β-Cells
Source: Sci Rep. 2016 May 16;6:25912. doi: 10.1038/srep25912 (PMC4867641; doi:10.1038/srep25912)
Supplement: Supplementary Information [file srep25912-s1.pdf]

Supplementary information

Potentiation of Glucose-stimulated Insulin Secretion by the GPR40–PLC–TRPC Pathway in Pancreatic β-Cells

Authors: Hodaka Yamada[1,*], Masashi Yoshida[1,*], Kiyonori Ito[1], Katsuya Dezaki[2], Toshihiko Yada[2], San-e Ishikawa[1], Masafumi Kakei[1]

[1]First Department of Comprehensive Medicine, Jichi Medical University Saitama Medical Center, Amanuma, Omiya 1-847, Saitama 330-8503, Japan

[2]Division of Integrative Physiology, Department of physiology, Jichi Medical University School of Medicine, Yakushiji 3311-1, Shimotsuke, Tochigi 329-0498, Japan

*These authors contributed equally to this work.

Supplementary Figure 1.

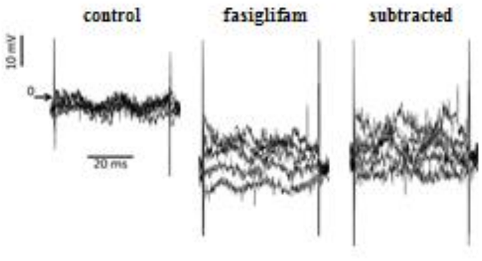

Supplementary Figure 1. Increases in NSCC current by fasiglifam. Perforated whole-cell clamp experiment performed in the absence (control) and presence of 10 μM fasiglifam showed that fasiglifam increased inward currents (see subtracted currents that were current components increased by fasiglifam). Original current traces were evoked by voltage steps of 10 mV increment from -80 mV to -40 mV with 10 sec interval. The holding potential was -70 mV and 100 μM tolbutamide was present throughout the current records.

Supplementary Figure 2.

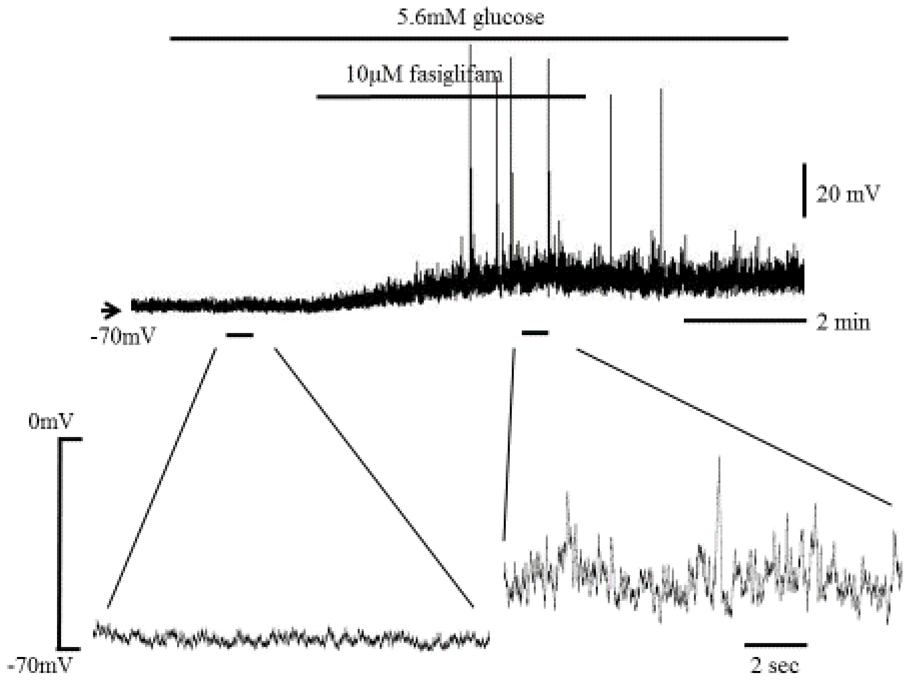

Supplementary Figure 2. Fsiglifam depolarized resting membrane potential.

The membrane potential was recorded at 5.6 mM glucose. The plasma membrane was depolarized upon superfusion with 10 μM fasiglifam. Firing profiles in specified periods at the top trace were shown in an expanded timescale below.

Supplementary Figure 3.

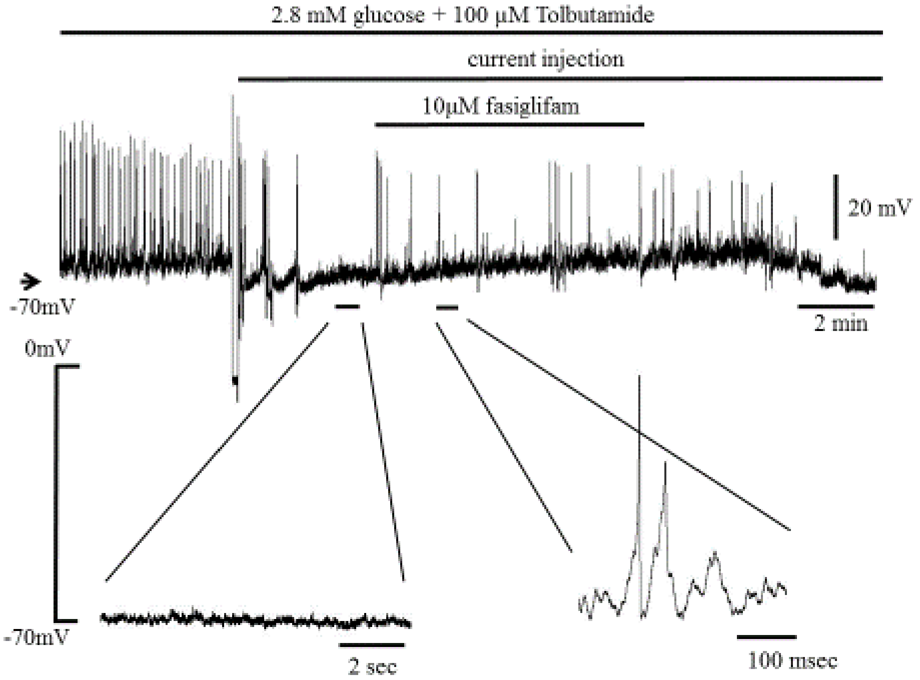

Supplementary Figure 3. Fsiglifam activated electrical activity after injection of hyperpolarizing current in the presence of 100 μM tolbutamide.

The membrane potential recorded in the presence of 100 μM tolbutamide and 2.8 mM glucose showed action-potential firing. After current was injected to hyperpolarize the membrane, action potentials were inhibited (see the left-side inset). The external solution was changed to that containing 10 μM fasiglifam. The membrane was depolarized again and firing of action potentials was induced (see the right-side trace below). These results suggest fasiglifam-evoked membrane depolarization independently of KATP-channel activity. Time-expanded traces corresponding to the indicated periods at the top trace were showed.

Supplementary Figure 4.

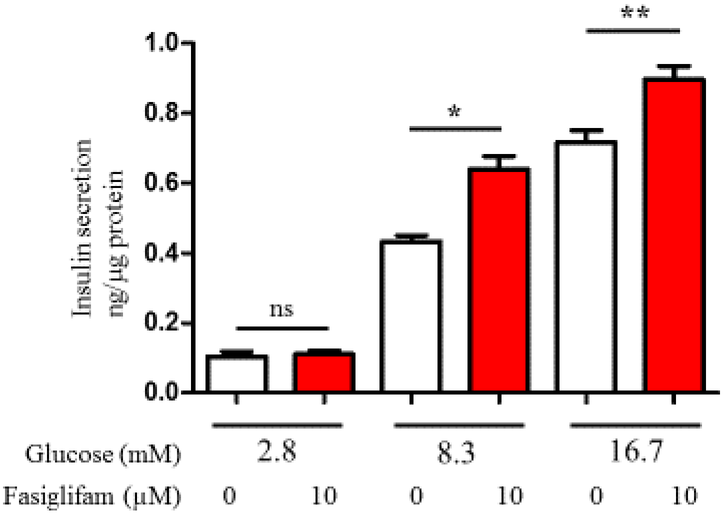

Supplementary Figure 4. Insulin secretion responses normalized by protein content (ng/µg protein) in rat islets. Each batch containing size-matched 10 rat islets was incubated one hour to evoke insulin secretion. Protein content of 10 islets was measured by BCA Protein Assay Kit (Thermo Fisher) according to an instruction of the manufacture. The number of data points was five. *P < 0.05 by unpaired t-test between with or without fasiglifam an indicated glucose level. ns; not significant.

Supplementary Figure 5.

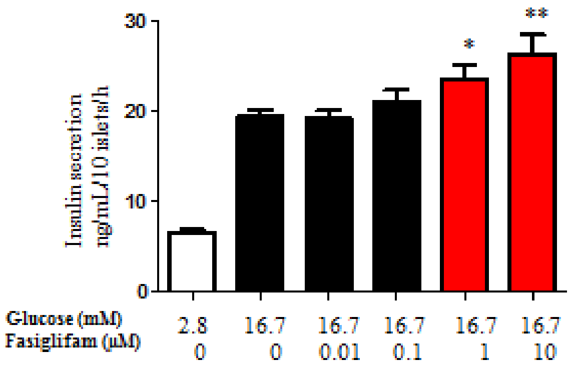

Supplementary Figure 5. Dose-dependent increase in insulin secretion from rat islets by fasiglifam. Insulin secretion was measured at indicated glucose and fasiglifam concentrations in the figure. The number of data points was from five to seven. *P < 0.05 vs. 16.7 mM glucose by unpaired t-test. Fasiglifam potentiated insulin secretion at 1 and 10 μM fasiglifam.

Supplementary Figure 6.

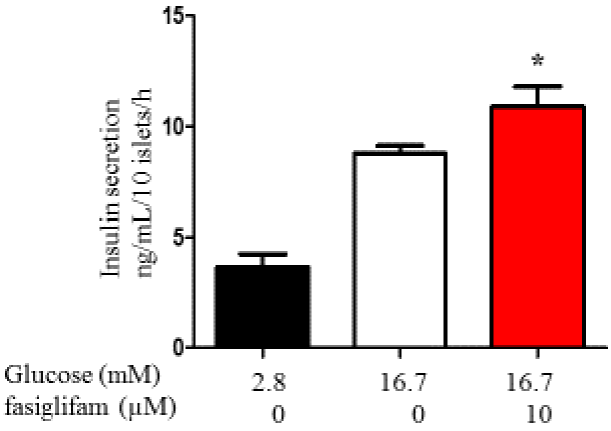

Supplementary Figure 6. Insulin secretion from mouse islets stimulated by glucose and fasiglifam. Insulin secretion was measured by mouse insulin ELISA kit (Morinaga). The number of data points was five to nine. *P < 0.05 vs. 16.7 mM glucose by unpaired t-test.

Supplementary Figure 7.

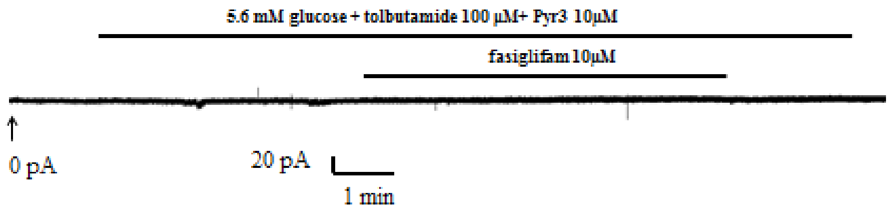

Supplementary Figure 7. Original current trace showing the effects of 10 µM pyrazole-3 (Pyr3). Pyr3 prevented current increase induced by fasiglifam in the presence of 5.6 mM glucose and 100 µM tolbutamide. The holding potential was held at −70 mV. Arrow indicates zero current level. This result was from a rat beta cell.

Supplementary Figure 8.

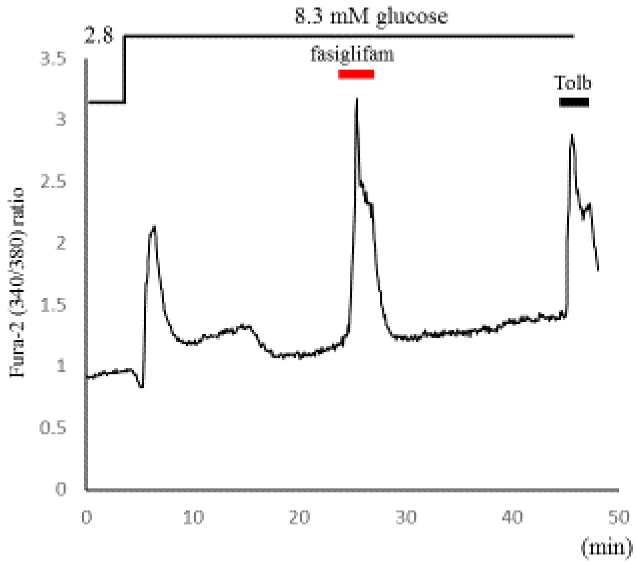

Supplementary Figure 8. Fasiglifam (10 μM) increased intracellular Ca^{2+} concentration ($[Ca^{2+}]_i$) in rat pancreatic beta cells at 8.3 mM glucose as revealed in Fig. 4 of which results from mice. At the end of the experiment, 100 μM tolbutamide (Tolb) was added to confirm that the responsive cells were beta cells.

Supplementary Figure 9.

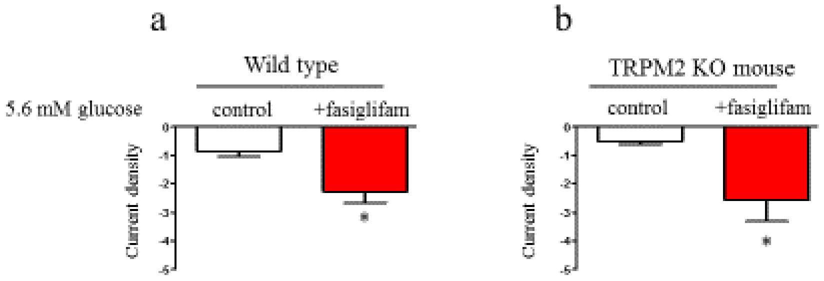

Supplementary Figure 9. Fasiglifam equally increased the nonselective cation channel (NSCC) current in beta cells from C57BL/6J mice (a) and TRPM2-knockout mice (b) at 5.6 mM glucose. The membrane potential was held at −70 mV in the presence of 100 μM tolbutamide. The number of data points was five. *P < 0.05 vs. control by paired t-test.

Supplementary Figure 10.

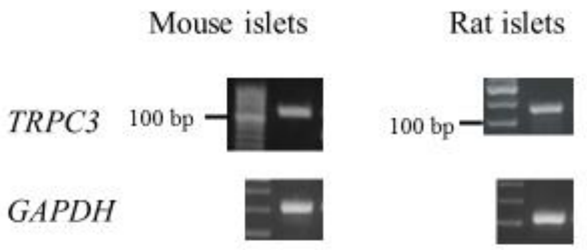

Supplementary Figure 10. Gene expression of mouse *TRPC3* and rat *TRPC3* in islets. The *TRP3* gene was expressed in rodent islet (RT-PCR). The *GAPDH* was used as internal control (see supplementary method). RNA was isolated from rat and mouse islets with Direct-zol™ RNA MiniPrep (ZYMO RESEARCH). Complementary DNA was made with ReverTra Ace qPCR RT Master Mix (TOYOBO). RT-PCR was performed with the LightCycler system and KOD SYBR qPCR Mix (TOYOBO) according to the manufacturer's instructions, and then subjected 2% agarose gel electrophoresis. The following primers were used (Sigma–Aldrich):

Rat *TRPC3*, forward (5'-3') CTGGCCAACATAGAGAAGGAGT, reverse (5'-3') CACCGATTCCAGATCTCCAT;

Mouse *TRPC3*, forward (5'-3') TTCAAGACTTTGTTTTGGTCCAT, reverse (5'-3') AGCAATTAGCATGTTGAGTAAAACG.
